# Acceptable risks of treatments to prevent rheumatoid arthritis among first-degree relatives: demographic and psychological predictors of risk tolerance

**DOI:** 10.1136/rmdopen-2022-002593

**Published:** 2022-12-13

**Authors:** Gwenda Simons, Ellen M Janssen, Jorien Veldwijk, Rachael L DiSantostefano, Matthias Englbrecht, Christine Radawski, Larissa Valor-Méndez, Jennifer H Humphreys, Ian N Bruce, Brett Hauber, Karim Raza, Marie Falahee

**Affiliations:** 1 Rheumatology Research Group, Institute of Inflammation and Ageing, University of Birmingham, Birmingham, UK; 2 Janssen Research and Development, Titusville, New Jersey, USA; 3 Erasmus School of Health Policy and Management and Erasmus Choice Modelling Centre, Erasmus University Rotterdam, Rotterdam, The Netherlands; 4 Julius Center for Health Sciences and Primary Care, University Medical Center Utrecht, Utrecht University, Utrecht, The Netherlands; 5 Freelance Data Scientist, Eckental, Germany; 6 Eli Lilly and Company, Indianapolis, Indiana, USA; 7 Department of Internal Medicine and Institute for Clinical Immunology, Friedrich-Alexander-University Erlangen-Nürnberg and Universitätsklinikum, Erlangen, Germany; 8 Centre for Epidemiology Versus Arthritis, Division of Musculoskeletal and Dermatological Sciences, School of Biological Sciences, Faculty of Biology, Medicine and Health, The University of Manchester, Manchester, UK; 9 Kellgren Centre for Rheumatology, Manchester University NHS Foundation Trust, Manchester, UK; 10 NIHR Manchester Biomedical Research Centre, Manchester University NHS Foundation Trust, Manchester Academic Health Science Centre, Manchester, UK; 11 Pfizer, New York, New York, USA; 12 Department of Rheumatology, Sandwell and West Birmingham NHS Trust, Birmingham, UK; 13 Research into Inflammatory Arthritis Centre Versus Arthritis and MRC-Versus Arthritis Centre for Musculoskeletal Ageing Research, University of Birmingham, Birmingham, UK

**Keywords:** Rheumatoid Arthritis, Antirheumatic Agents, Psychology

## Abstract

**Objectives:**

To quantify tolerance to risks of preventive treatments among first-degree relatives (FDRs) of patients with rheumatoid arthritis (RA).

**Methods:**

Preventive treatments for RA are under investigation. In a preference survey, adult FDRs assumed a 60% chance of developing RA within 2 years and made choices between no treatment and hypothetical preventive treatment options with a fixed level of benefit (reduction in chance of developing RA from 60% to 20%) and varying levels of risks. Using a probabilistic threshold technique, each risk was increased or decreased until participants switched their choice. Perceived risk of RA, health literacy, numeracy, Brief Illness Perception Questionnaire and Beliefs about Medicines Questionnaire-General were also assessed. Maximum acceptable risk (MAR) was summarised using descriptive statistics. Associations between MARs and participants’ characteristics were assessed using interval regression with effects coding.

**Results:**

289 FDRs (80 male) responded. The mean MAR for a 40% reduction in chance of developing RA was 29.08% risk of mild side effects, 9.09% risk of serious infection and 0.85% risk of a serious side effect. Participants aged over 60 years were less tolerant of serious infection risk (mean MAR ±2.06%) than younger participants. Risk of mild side effects was less acceptable to participants who perceived higher likelihood of developing RA (mean MAR ±3.34%) and more acceptable to those believing that if they developed RA it would last for a long time (mean MAR ±4.44%).

**Conclusions:**

Age, perceived chance of developing RA and perceived duration of RA were associated with tolerance to some risks of preventive RA therapy.

**WHAT IS ALREADY KNOWN ON THIS TOPIC**

- Preventive interventions for rheumatoid arthritis (RA) could reduce patient burden and societal cost at scale, but few studies have quantified the preferences of at-risk groups for risks and benefits of treatments to reduce the risk of RA.

**WHAT THIS STUDY ADDS**

- This study quantified the degree of treatment risk that first-degree relatives of patients with RA would accept for a given benefit and assessed participant characteristics that account for variability in risk tolerance.

**HOW THIS STUDY MIGHT AFFECT RESEARCH, PRACTICE OR POLICY**

- The findings of this study are informative for the development of informational resources for those at-risk of developing RA and also for decision-making throughout the development of preventive treatments for RA.

## Introduction

Rheumatoid arthritis (RA) is a chronic inflammatory condition for which long-term treatment with disease-modifying antirheumatic drugs (DMARDs) is usually required. Early treatment of RA is associated with improved outcomes and is a key element of treatment guidelines.^1^ Increased research focus on the early phases of RA development has led to the recognition that biomarkers and symptoms associated with RA development may precede the onset of RA, and several at-risk phases where preventive intervention may be possible have been identified.^2^

Prevention of RA could result in reduced pain and disability for patients, as well as considerable reduction in healthcare costs.^3^ Several trials of time-limited pharmaceutical interventions to prevent RA development in at-risk groups have been completed or are ongoing,^4–8^ including trials in asymptomatic first-degree relatives (FDRs) of patients with RA,^8^ who are approximately four times more likely to develop RA than members of the general public.^9^

A recent trial of atorvastatin to prevent RA development in patients with seropositive arthralgia was prematurely discontinued as a result of the unwillingness of prospective trial participants to take part due to concerns around the potential for benefit, treatment side effects and the burden of trial participation.^10 11^ A EULAR task force on conducting clinical trials and observational studies in individuals at risk of RA has subsequently highlighted the need to address difficulties in recruitment to RA prevention studies. Emphasis was placed on the importance of understanding what interventional strategies are acceptable to those at risk to inform the design of future prevention trials and informational resources for participants to support trial recruitment and clinical translation.^12 13^ Furthermore, systematically collected information about patient preferences is increasingly valued for stakeholder decision-making during the development and regulation of new medical products.^14 15^

While preventive interventions for RA could reduce patient burden and societal cost at scale, few studies have quantified the preferences of at-risk groups for risks and benefits of treatments to reduce the chance of developing RA. Previous qualitative studies have found that the acceptability of preventive approaches for RA is influenced by perceptions of treatment effectiveness and harms,^16^ but there are limited examples of studies to quantify the relative importance of treatment attributes, benefit–risk trade-offs and preference heterogeneity.^17^ One study eliciting preferences for preventive treatments for RA which used profile-case best-worst scaling^18^ had a relatively small sample size and did not report acceptable benefit–risk trade-offs. A small number of quantitative studies have employed discrete choice experiments (DCEs),^19–21^ which require large sample sizes to estimate reliable models and to account for both acceptability of treatment risks and heterogeneity among preferences. Preference heterogeneity in a DCE study of 288 self-reported FDRs was not explained by participant characteristics.^19^

Since public perceptions of RA are often incorrect,^22^ recruitment of confirmed rather than self-reported FDRs indirectly through patients attending outpatient clinics offers greater certainty around the at-risk status of participants, but with potentially inefficient recruitment rates.^23^ The objectives of this study were to quantify risk tolerance to treatments to prevent RA among a sample of confirmed FDRs and to assess the associations between variability in risk tolerance and participant characteristics. Probabilistic threshold technique (PTT)^24^ has been used to quantify treatment preferences successfully with relatively small sample sizes across a range of healthcare contexts.^25^ As this method allows direct rather than estimated assessment of treatment benefit–risk trade-offs, it is particularly suitable for assessing associations between preferences and participant characteristics, and has been identified as being likely to meet most decision-makers’ needs during all stages of the medical product life-cycle.^26^ Therefore, the current study uses PTT to assess confirmed FDRs’ preferences for preventive treatments for RA.

## Methods

This study is part of a case study for the Innovative Medicines Initiative project ‘PREFER’ (Patient Preferences in Benefit-Risk Assessments during the Medical Product Lifecycle), which aimed to develop evidence-based recommendations on how and when preference studies can inform decision-making during drug development.^14^ The case study protocol^27^ and other results from the case study^21^ have been published previously.

### Participant identification and recruitment

Eligible participants were aged 18 years or older, without a diagnosis of RA, provided informed consent and were FDRs of patients with RA. Patients with a clinical diagnosis of RA were approached in person or by mail via rheumatology outpatient clinics in West Midlands, UK, between 26 November 2020 and 22 March 2021 and were asked to invite one or more FDRs to take part in an online survey study. FDRs recruited similarly via rheumatology clinics who were enrolled in a UK prospective observational cohort (PRe-clinical EValuation of Novel Targets in RA: PREVeNT-RA) were also invited via direct email. Surveys (hosted by SurveyEngine) were completed anonymously. Participants were offered £5 online gift voucher as an incentive.

### Procedure

PTT^24^ was used to elicit the maximum acceptable treatment risk (MAR) that participants would accept for a treatment to reduce their risk of developing RA. This method has been identified as an efficient tool to collect quantitative information about patient treatment preferences in healthcare settings, including trade-offs between treatment attributes (eg, risks and benefits).^26^ The MAR is the highest level of risk that patients would tolerate in exchange for the benefit (ie, improvement in effectiveness) offered by the treatment.

**Figure 1:**
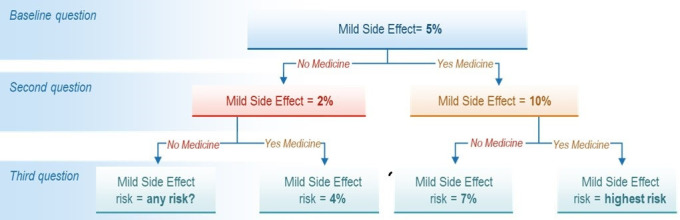
Schematic example of probabilistic threshold technique for the chance of mild side effects.

Participants were asked to imagine that (1) they were experiencing joint pain that was impacting on their daily activities and had received test results indicating their chance of developing RA in the next 2 years was 60%; (2) their doctor has suggested they consider taking a treatment to reduce their chance; and (3) the duration of preventive treatment would be for 1 year. Participants were first asked to choose between the status quo (no preventive treatment: 60% chance of developing RA and no risk of side effects) or a hypothetical treatment with a fixed level of benefit (reduction in the chance of developing RA from 60% to 20%), and baseline levels of the three treatment-related risks. For each risk under consideration, the level of that risk was systematically varied (either upward or downward, depending on the participant’s initial response) while holding the levels of the other two risks at baseline, until participants changed their choice. Baseline values for the risks in the PTT were 5%, 2% and 0.02% for mild side effects, serious infection and serious side effects, respectively (see figure 1 for a schematic representation). This procedure was repeated for each of the remaining two risks (serious infection and serious side effect) to define the risk interval for each participant for each risk presented in the survey.

The selection of 60% risk of RA development in the treatment scenario that participants were asked to assume in each choice task was informed by the range that is currently predictable using an existing algorithm for prediction of RA development in patients with seropositive arthralgia.^28^ It was further informed by extensive consultation with clinical experts and patient research partners about the level of risk at which preventive treatment with DMARDs would be appropriate and acceptable to this at-risk group, who are the population of interest in most current or previous clinical studies of treatment to reduce risk of RA. The selection of treatment attributes included in the PTT was informed by a literature review,^17^ a qualitative study and an attribute ranking survey.^27^ The final attributes (table 1) were agreed upon by an international team of clinical researchers, consultant rheumatologists, preference elicitation experts and patient partners. Attribute levels were estimated with input from clinical experts, including researchers leading the development and/or clinical trials of preventive treatments for RA. The text used to describe attributes and levels to participants is available in online supplemental material. The survey was codeveloped with patient partners and pretested in a convenience sample of FDRs and members of the general public (n=15) using ‘think-aloud’ interviews to refine user-friendliness and identify any programming errors. Participants in the pretesting exercise were paid £20 in shopping vouchers.

10.1136/rmdopen-2022-002593.supp1 Supplementary data

**Table 1:** Attributes and levels of treatment options

Treatment attribute	Levels describing no treatment option (%)	Levels describing treatment option (%)
Chance of developing rheumatoid arthritis	60	20
Chance of mild side effects	0	2, 4, **5**, 7 or 10*
Chance of a serious infection due to treatment	0	1, 1.5, **2**, 3 or 5*
Chance of a serious side effect that is potentially irreversible	0	0.001, 0.01, **0.02**, 0.05 or 0.1*

*One by one, each treatment risk was systematically increased or decreased across these levels (depending on the participant’s initial response; baseline level indicated in bold type) until the participant switches choice (choosing no treatment, instead of a preventive treatment, or vice versa).

### Survey content

The survey included (1) background information about RA (included in online supplemental material); (2) questions to assess comprehension of background material; (3) introduction to the choice tasks and treatment attributes and levels (included in online supplemental material); (4) guided ‘walk-through’ choice task example; (5) warm-up choice tasks; and (6) the series of PTT choice tasks (example choice task included in online supplemental material). During completion of each choice task, participants could choose to view the explanation of each attribute and its levels (including icon arrays for the risk-related levels) using pop-up windows.

The survey also included measures of sociodemographic variables and the following: (1) the Single Item Health Literacy Screener,^29^ which assesses how often a participant needs assistance to read written information provided by a healthcare professional on a 5-point scale, with scores greater than 2 indicating some difficulty reading printed health-related material; (2) the three-item version of the Subjective Numeracy Scale,^30^ where each item is scored on a 6-point scale, with higher scores indicating stronger perceived numeracy; (3) the Beliefs about Medicines Questionnaire-General (BMQ-G),^31^ which consists of two subscales, both comprising the sum of four items with 5-point Likert scale responses—the General-Overuse subscale addresses respondents’ views about the way in which medicines are used by doctors, and the General-Harm subscale assesses their beliefs about the degree to which medicines are harmful, with lower scores indicating stronger agreement with statements that medicines are harmful/overused; (4) the Brief Illness Perception Questionnaire (B-IPQ),^32 33^ adapted for individuals without the relevant disease^34^ and which assesses participants’ perceptions of what it would be like to have RA in relation to eight subscales (consequences, timeline, personal control, treatment control, identity, concern, understanding and emotional response), each of which is scored on an 11-point scale; and (5) participants also assessed their lifetime risk of developing RA using a 5-item Likert-type scale (‘very unlikely’ to ‘very likely’). Selection of the included measures was informed by a systematic review^35^ and related consensus study and recommendations,^36^ with input from patient partners and clinical experts. On completion of the survey, all respondents were provided with an informationresource about RA and risk factors for RA which had been developed by an international team of patient partners, rheumatologists and researchers as part of a previous project.^37^

### Sample size and data analysis

There is no specific power calculation to determine sample size in PTT studies without knowing the expected threshold value a priori. Most PTT studies are conducted with 100 or fewer respondents, and substantially smaller samples (between 20 and 42 respondents) have been used successfully in previous studies.^38–41^ The outcomes of interest were risk equivalents (MAR) for each treatment risk attribute. Results were considered statistically significant if p≤0.05. Analyses were carried out using SPSS V.27.0 and R V.3.6.1. Only fully completed surveys were included in the analysis.

The series of PTT questions resulted in a threshold interval for each treatment risk that represented the risk level each participant was willing to accept in exchange for the benefit (reduction in chance of developing RA from 60% to 20%). These data were analysed using descriptive statistics and interval regression models, in which the data are interval-censored because the threshold falls within an interval with fixed endpoints. For each risk, an interval regression model was fitted using a tobit model to account for the fact that the interval has both a fixed upper bound, resulting in left-censored data, and a fixed lower bound, resulting in right-censored data.^42^ Participant characteristics were included as categorical covariates to assess their association with risk tolerance: age (below 60 vs 60 years old and above), education (below graduate level vs graduate and above), health literacy (low (need help ‘sometimes’, ‘often’ or ‘always’) vs high (need help ‘occasionally’ or ‘never’)), numeracy (low (average numeracy score 3 or below) vs high (average numeracy score 4 or above)), B-IPQ subscales (below median score vs above median score), BMQ-G subscales (below median score vs above median score) and perceived risk of RA (very unlikely, unlikely and neutral vs likely or very likely).

$$\begin{array}{ll} MAR_{r} & =\alpha_{r}+\beta_{1}Age_{i}+\beta_{2}Education_{i}+\beta_{3}HealthLiteracy_{i}\\ & +\beta_{4}Numeracy_{i}+\beta_{5}BIPQsubscales_{i}+\beta_{6}BMQGsubscales\\ & +\beta_{7}PerceivedRisk_{i}+\varepsilon_{r} \end{array}$$

All covariates were effects-coded in the regression model. As a result, the coefficients (βs) for each of the categories of the covariates always sum up to 1 (eg, $\beta_{1}Age_{below60}+\beta_{1}Age_{above60}=0$). The coefficients for the covariates represent the effect of meeting that participant characteristic on the mean MAR.

### Patient and public involvement

An international panel of eight patient research partners (based in the UK (n=4), Germany (n=2), the Netherlands (n=1) and Sweden (n=1)) actively contributed to the development of research objectives; a formative qualitative study, selection of treatment attributes and levels included in choice tasks; development of choice task scenario; survey content and participant information; choice of additional questionnaires included in the survey and survey pretesting; and coauthorship of a public summary of study findings. Examples of changes made as a result of their contribution include additional material in the survey background material describing examples of the impact of early RA symptoms on daily activities, removal of a figure illustrating joint erosion associated with RA, increase to the baseline chance of developing RA that participants were asked to assume in the choice task, removal of additional measures of psychological constructs to reduce participant burden and rewording of the survey content to improve readability.

## Results

Participants’ demographic characteristics are described in table 2. The survey was completed by 289 FDRs, of whom 80 were male. Of the participants, 94% described their ethnicity as white, 67% were educated to graduate level or above, and 47% were working in full-time employment. Responses to measures of cognitive abilities and illness/medication beliefs are summarised in table 3.

**Table 2:** Demographic characteristics of first-degree relatives (N=289)

		Frequency (%)
Gender	Male	80 (27.7)
	Female	209 (72.3)
Age	18–29	23 (8.0)
	30–39	45 (15.6)
	40–49	75 (26.0)
	50–59	86 (29.8)
	60–69	47 (16.3)
	70 and over	13 (4.5)
Ethnicity	Asian	10 (3.5)
	Black African Caribbean	1 (0.3)
	White	271 (93.8)
	Other	7 (2.4)
Highest level of education	None	1 (0.3)
	Primary school	0
	Secondary school	41 (14.2)
	Sixth form	48 (16.6)
	Degree or vocational	123 (42.6)
	Postgraduate	72 (24.9)
	Other	4 (1.4)
Employment	Employed part-time	51 (17.8)
	Employed full-time	135 (46.7)
	Self-employed part-time	16 (5.5)
	Self-employed full-time	18 (6.2)
	Student part-time	0
	Student full-time	12 (4.2)
	Home maker	5 (1.7)
	Unemployed	3 (1.0)
	Not working due to disability	5 (1.7)
	Retired	44 (15.2)
	Other	6 (2.1)

**Table 3:** Participants’ perceived risk, health literacy, subjective numeracy and responses to the B-IPQ and BMQ subscales (N=289)

Variable	Mean (SD)	Median (IQR)
Perceived likelihood of developing rheumatoid arthritis in your lifetime (1: very unlikely, 5: very likely)	3.50 (0.86)	4.00 (3–4)
Single Item Literacy Screener (1: always need help to understand written information from doctor, 5: never need help)	4.85 (0.59)	5.00 (5–5)
Three-Item Subjective Numeracy Scale total score (3: lowest numeracy score, 18: highest numeracy score)	15.00 (3.03)	16.00 (14–17.5)
BMQ-General-Harm subscale total score (4: most agreement that medicines are harmful, 20: least agreement that medicines are harmful)	16.00 (2.39)	16.00 (14–18)
BMQ-General-Overuse subscale total score (4: most agreement that medicines are overused, 20: least agreement that medicines are overused)	13.79 (2.80)	14.00 (12–16)
B-IPQ1 (consequences): if you were to develop rheumatoid arthritis, how much do you think it would affect your life? (0: not affect me at all, 10: severely affect my life)	8.26 (1.49)	8.00 (7–10)
B-IPQ2 (timeline): if you were to develop rheumatoid arthritis, how long do you think it would continue? (0: a very short time, 10: forever)	9.67 (0.92)	10.00 (10–10)
B-IP3 (personal control): if you were to develop rheumatoid arthritis, how much control do you think you would have over it? (0: absolutely no control, 10: extreme amount of control)	4.28 (2.22)	5.00 (3–6)
B-IPQ4 (treatment control): if you were to develop rheumatoid arthritis, how much do you think your treatment would help it? (0: not at all helpful, 10: extremely helpful)	7.12 (1.84)	7.00 (6–8)
B-IPQ5 (identity): if you were to develop rheumatoid arthritis, how much do you think you would experience symptoms from it? (0: no symptoms at all, 10: many severe symptoms)	7.67 (1.49)	8.00 (7–9)
B-IPQ6 (concern): if you were to develop rheumatoid arthritis, how concerned do you think you would be about it? (0: not at all concerned, 10: very concerned)	8.83 (1.52)	9.00 (8–10)
B-IPQ7 (coherence): if you were to develop rheumatoid arthritis, how well do you think you would understand it? (0: not understand at all, 10: understand very clearly)	8.2 (1.64)	8.00 (7–10)
B-IPQ8 (emotional representation): if you were to develop rheumatoid arthritis, how much do you think it would affect you emotionally? (0: not at all affected emotionally, 10: extremely affected emotionally)	7.54 (2.13)	8.00 (7–9)

B-IPQ, Brief Illness Perceptions Questionnaire; BMQ, Beliefs about Medicines Questionnaire.

**Figure 2:**
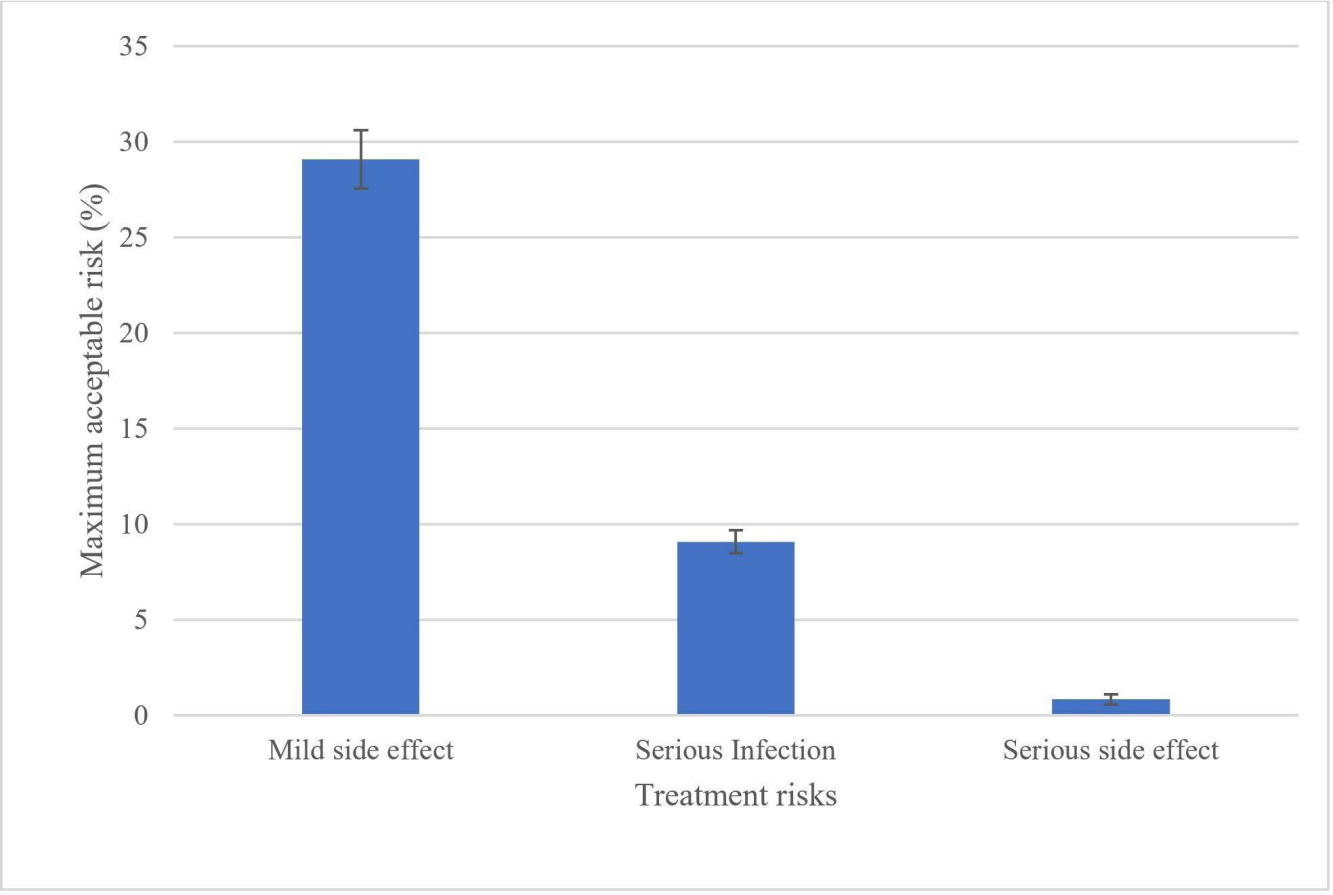
Maximum acceptable risk of mild side effects, serious infection and serious side effects for a reduction in risk of rheumatoid arthritis from 60% to 20%.

MARs in exchange for a reduction in chance of developing RA from 60% to 20% are summarised in figure 2 (see online supplemental material for the distribution of MAR intervals for each risk attribute). When not accounting for covariates, on average, participants would accept a 29% increase in risk of mild side effects, a 9% increase in risk of serious infection and less than 1% increase in risk of serious side effects.

When controlling for covariates, participants aged over 60 years were less tolerant of risk of serious infection (ΔMAR (SE): −2.06% (0.78)) than younger participants (ΔMAR (SE): +2.06% (0.78)). Risk of a mild side effect was less acceptable to participants who perceived they were likely/very likely to develop RA (ΔMAR (SE): −3.34% (1.55)) than to those who perceived themselves to be very unlikely/unlikely to develop RA/neutral (ΔMAR (SE): +3.34% (1.55)). Risk of a mild side effect was also more acceptable to participants who perceived that if they were to develop RA it would last for a longer time (Δ MAR (SE): +4.44% (2.20)) than those who perceived that RA would last for a shorter time (ΔMAR (SE): −4.44% (2.20)). Education level, health literacy, numeracy, all seven other B-IPQ subscales and both BMQ-G subscales did not predict participants’ treatment preferences. The results of the interval regression controlling for covariates are summarised in table 4.

**Table 4:** Interval regression results to predict MAR (N=289)

Treatment risks		Mild side effect		Serious infection		Serious side effect	
		Coefficient	SE	Coefficient	SE	Coefficient	SE
Mean MAR (%)		**21.73**	4.38	**8.93**	1.81	−0.28	0.80
Age	18–60	2.66	1.94	**2.06**	0.78	0.44	0.36
	60 or over	−2.66	1.94	−**2.06**	0.78	−0.44	0.36
Education	Below graduate	−0.37	1.68	−0.51	0.67	0.22	0.31
	Graduate or above	0.37	1.68	0.51	0.67	−0.22	0.31
Health literacy	Low	−1.72	3.81	0.81	1.60	−0.51	0.70
	High	1.72	3.81	−0.81	1.60	0.51	0.70
Subjective numeracy	Low	−0.69	2.25	0.81	0.89	−0.21	0.40
	High	0.69	2.25	−0.81	0.89	0.21	0.40
Brief Illness Perception Questionnaire subscales	Consequences −	−1.58	2.01	1.31	0.80	−0.13	0.37
	Consequences +	1.58	2.01	−1.31	0.80	0.13	0.37
	Timeline −	−**4.44**	2.20	−0.19	0.87	−0.01	0.40
	Timeline +	**4.44**	2.20	0.19	0.87	0.01	0.40
	Personal control −	−1.35	1.66	−0.41	0.65	−0.34	0.30
	Personal control +	1.35	1.66	0.41	0.65	0.34	0.30
	Treatment control −	1.72	1.59	0.47	0.62	0.03	0.29
	Treatment control +	−1.72	1.59	−0.47	0.62	−0.03	0.29
	Identity −	1.55	1.66	−0.82	0.66	−0.24	0.30
	Identity +	−1.55	1.66	0.82	0.66	0.24	0.30
	Concern −	0.71	1.92	−1.41	0.76	−0.38	0.35
	Concern +	−0.71	1.92	1.41	0.76	0.38	0.35
	Coherence −	0.34	1.56	−0.15	0.61	−0.35	0.28
	Coherence +	−0.34	1.56	0.15	0.61	0.35	0.28
	Emotion −	−0.59	1.71	−0.09	0.68	0.20	0.31
	Emotion +	0.59	1.71	0.09	0.68	−0.20	0.31
Beliefs about Medicines Questionnaire-General subscales	Harm −	−2.03	1.67	0.00	0.66	−0.26	0.30
	Harm +	2.03	1.67	0.00	0.66	0.26	0.30
	Overuse −	1.37	1.63	0.44	0.64	0.31	0.29
	Overuse +	−1.37	1.63	−0.44	0.64	−0.31	0.29
Perceived risk of RA	Very unlikely/unlikely/neutral	**3.34**	1.55	0.22	0.61	0.09	0.28
	Likely/very likely	−**3.34**	1.55	−0.22	0.61	−0.09	0.28

Boldface values denote estimated coefficients that are significant at the 5% level.

−, below median; +, above median.

MAR, maximum acceptable risk; RA, rheumatoid arthritis.

## Discussion

This is the first study to use PTT to quantify risk tolerance of at-risk individuals to preventive treatment of RA, addressing an evidence gap for research on preferences in this area identified in a recent systematic review study.^17^ Participants’ tolerance to specific treatment risks was explained by age, perceived risk of developing RA and one B-IPQ subscale (timeline), but not by health literacy, subjective numeracy, other illness perceptions or beliefs about medicines.

The role of perceived personal risk of developing RA in preferences of at-risk individuals for preventive treatments for RA aligns with a recent study that found perceived risk was associated with FDRs’ interest in predictive testing for RA.^43^ Baseline personal risk of developing RA also affected preferences for preventive treatments for RA in a pilot best-worst scaling study of FDRs.^18^ Therefore, it is important for further studies to understand drivers of perceived risk of developing RA in at-risk groups. In the absence of additional risk factors, FDRs’ lifetime chance of developing RA is approximately 4%.^9^ The fact that the perceived chance of developing RA was high among this sample of FDRs highlights the need to develop effective, balanced risk communication tools for those considering taking part in RA prevention studies^12^ and risk management strategies. It should be noted that, in the survey instrument used in the current study, perceived personal chance of developing RA was assessed after the choice tasks in which participants were asked to assume a hypothetical 60% chance of developing RA in the next 2 years based on positive blood test results. This might, therefore, have increased their subsequent assessment of perceived chance of developing RA.

Measures of health literacy and numeracy have explained heterogeneity in treatment preferences in other disease contexts,^35^ but not in this study. This could be due to relatively high levels of literacy and numeracy in the sample and/or a similar understanding of concepts in the survey across participants. It is also counterintuitive that all but one measure of perceptions about RA and beliefs about medicines did not explain preferences in this study, although evidence relating to the role of these constructs in treatment preferences is limited.^35^ Nevertheless, beliefs around illness and treatment are known to affect health behaviours including treatment adherence in RA.^44 45^ It could be that in this sample there is insufficient heterogeneity in these constructs and/or in participants’ treatment preferences to enable detection of an association, or that the background information provided to participants reduced variation in perceptions about RA and the necessity of medication. Further investigation is needed to understand the impact of interventions to modify illness perceptions on preferences for preventive treatments for RA and on treatment preferences more generally.^46^ It is also possible that perceptions of illness and treatments are more relevant for preferences for treatment of established disease than they are for preferences for treatments to reduce risk of disease development. Participants’ lifestyle, plans regarding future pregnancy (in them/their partner), and experiences of other diseases and associated treatment were not assessed in the current study, but could also account for variability in tolerance to treatment risks in this context. Further investigation is needed to gain a comprehensive understanding of additional factors associated with preference heterogeneity.

All preference studies of preventive treatment for RA conducted to date have focused on the effectiveness of treatments to reduce risk of RA as the relevant treatment benefit. Further investigation is warranted to establish preferences for preventive treatment benefits other than effectiveness to reduce risk, including delay in RA development and impact on early symptoms that may precede the diagnosis of RA.

In the present study, we chose to focus on a treatment scenario for a symptomatic at-risk individual, corresponding to antibody-positive clinically suspect arthralgia, since this group is the population of interest in most existing clinical trials of preventive treatments for RA.^6 7^ To our knowledge, only one interventional trial is currently recruiting asymptomatic individuals: the Strategy to Prevent the Onset of Clinically Apparent RA (StopRA) study.^8^ No studies to date have quantified the extent to which at-risk individuals would accept risks associated with preventive interventions in the absence of symptoms, and further investigation is needed to address this important topic.

The present study was designed to inform the development of pharmacological products to reduce the risk of RA development since several clinical trials have assessed the ability of drugs to reduce RA development. Preventive behavioural/lifestyle interventions are also likely to be effective and may be preferred by patients.^13^ The efficacy of lifestyle approaches to reduce the risk of RA development in at-risk groups and patient preferences for such an approach are important topics for further study.^47^

### Strengths and limitations

Strengths of this study include the large sample size and the extensive input from clinical researchers, preference elicitation experts and patient research partners. The treatment attributes and the levels included were based on extensive literature review, qualitative investigation, ranking surveys and expert consultation, in line with best practice for preference studies.

Another important strength is that FDRs were recruited via probands with a confirmed diagnosis of RA. The only other quantitative study to use stated choice methods to elicit FDRs’ preferences for preventive treatment of RA that had a comparable sample size recruited self-reported FDRs through an online recruitment platform.^19^ Reliance on self-reported FDR status is limiting, since public perceptions of RA are often incorrect and RA is often confused with other common conditions such as osteoarthritis.^22^ Therefore, it is likely that the samples of FDRs in previous studies may not be representative of the target population.

However, recruitment of FDRs via patients with RA introduces opportunities for sample bias both at the participant level and the proband level. It is possible that FDRs who perceived their risk of developing RA to be higher were more likely to take part in this study. Patients’ decision-making around which, if any, relatives to invite to take part in the study is likely to be based on a subjective assessment of their likely interest and engagement with the subject matter.^48^ While proband-led recruitment in prevention studies maximises participant privacy, approaches that allow the clinical research team to directly access FDRs may result in recruitment of larger, more heterogeneous samples.^49^

All stated choice studies ask participants to make hypothetical choices between treatment options. In this study, participants were asked to assume a hypothetically elevated risk of RA. Previous research has highlighted common public misperceptions around RA that are likely to impact beliefs around the need for preventive treatment and associated decision-making.^50^ We addressed this possibility by close collaboration with patient partners during the development of background information for survey participants and the inclusion of comprehension questions to check participant understanding of this material. Further research is needed to directly assess preferences of individuals with a high risk of RA, including those with RA-related autoantibodies and inflammatory-type joint symptoms.

Finally, data collection occurred within the year following the outbreak of the COVID-19 pandemic amidst vaccination campaigns and media coverage of the potential role of immunomodulatory agents used to treat RA in the treatment of COVID-19. This context is likely to have affected public perceptions of treatment risks/benefits in general and may not be generalisable to prepandemic preferences.

## Conclusions

FDRs of patients with RA asked to assume a 60% chance of developing RA within 2 years are willing to accept risks of treatment to reduce their chance of developing RA to 20%. Mild side effects were more acceptable than serious infection, which in turn was more acceptable than serious side effects. Tolerance to specific treatment risks was associated with age, perceived risk of developing RA and beliefs about how long RA would last if it were to develop.

## Data Availability

Data are available upon reasonable request.
