# Correction: Acceptable risks of treatments to prevent rheumatoid arthritis among first-degree relatives: demographic and psychological predictors of risk tolerance

**DOI:** 10.1136/rmdopen-2022-002593corr1

**Published:** 2023-01-24

Simons G, Janssen EM, Veldwijk J, *et al*. Acceptable risks of treatments to prevent rheumatoid arthritis among first-degree relatives: demographic and psychological predictors of risk tolerance. *RMD Open* 2022;8:e002593. doi: 10.1136/rmdopen-2022-002593

The funding statement for this article has been updated.

