# Supplementary material for: Acceptable risks of treatments to prevent rheumatoid arthritis among first-degree relatives: demographic and psychological predictors of risk tolerance
Source: RMD Open. 2022 Dec 13;8(2):e002593. doi: 10.1136/rmdopen-2022-002593 (PMC9748990; doi:10.1136/rmdopen-2022-002593)
Supplement: Supplementary data [file rmdopen-2022-002593supp001.pdf]

Acceptable risks of treatments to prevent rheumatoid arthritis amongst first-degree relatives: demographic and psychological predictors of risk tolerances (Simons et al)

Supplemental Material

Description of survey content

The survey included:

[i] demographic questions

[ii] background information about RA (see below)

[iii] multiple choice questions to assess comprehension of background material

[iv] introduction to the choice tasks and treatment attributes and levels (see below)

[v] guided 'walk-through' choice task example

[v] warm-up choice tasks

[vi] the series of PTT choice tasks (see below for example). During completion of each choice task, participants could choose to view the explanation of each attribute and its levels (including icon arrays for the risk-related levels) using pop-up windows.

[vii] The Single Item Health Literacy Screener (1)

[viii] The three-item version of the Subjective Numeracy Scale (2)

[ix] The Beliefs about Medicines Questionnaire-General (BMQ-G)(3)

[x] The Brief Illness Perception Questionnaire (B-IPQ)(4, 5) adapted for individuals without the relevant disease (6)

[xi] Likert-type scale to assess perceived likelihood of developing RA

[xii] Likert type scales to assess how easy the choice tasks were to understand, and how easy they were to complete

[xiii] Link to an information leaflet about risk of RA for first degree relatives of RA patients which had been developed by an international team of patient partners, rheumatologists and researchers as part of a previous project.(9)

Survey introduction to rheumatoid arthritis

Rheumatoid arthritis is a long-term condition that causes severe pain, swelling and stiffness in the joints. The symptoms usually affect the hands, feet and wrists, but can affect other joints and other parts of the body too. Some people with rheumatoid arthritis also experience symptoms such as fatigue and depression. Rheumatoid arthritis is often confused with osteoarthritis which is generally caused by wear and failed repair of the joints and affects many people as they get older. Rheumatoid arthritis is not a natural part of ageing, it is an autoimmune disease. This means your immune system – which usually fights infection – harms the cells in your joints making the joints swollen, stiff and painful. Over time, if untreated, this can damage the joint, causing deformity and loss of function. When uncontrolled, rheumatoid arthritis can have a severe impact on your ability to perform everyday activities. For example, you might find it difficult to close shirt buttons, lift a kettle, or perform activities such as turning keys, opening lids or packaging, walking, cycling and driving. The symptoms might even affect your ability to continue working. Having rheumatoid arthritis may further lead to other conditions such as inflammation of other parts of the body, including the lungs, heart and eyes. The life expectancy of people with rheumatoid arthritis is typically reduced by about ten years because of increased likelihood of conditions such as heart attacks and infections.

Once people have developed rheumatoid arthritis it currently cannot be cured. Although there are effective therapies to reduce symptoms, these therapies usually have to be taken in the long term, and can have a range of side effects, including serious infections, allergic reactions and an increased chance of certain types of cancers. Unfortunately, some people do not respond to existing therapies or may have to try several different therapies before they find the one that is effective for them.

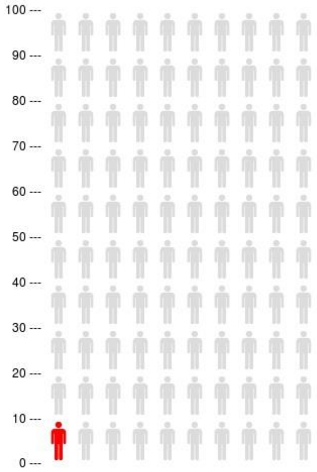

Rheumatoid arthritis usually begins when people are between 40 and 60 years old but can begin at other ages. Out of every 100 members of the general public, one person will develop rheumatoid arthritis, as illustrated by the single red figure in this picture. The 99 uncoloured figures represent people who will not develop rheumatoid arthritis.

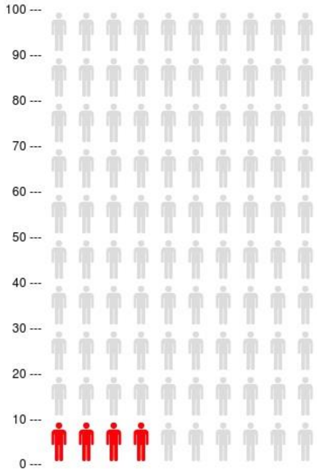

However, out of every 100 people who have a parent or sibling with rheumatoid arthritis, four people will develop rheumatoid arthritis, as illustrated by the four red figures in this picture. The 96 uncoloured figures represent people with a parent or sibling with rheumatoid arthritis who will not develop rheumatoid arthritis.

Some aspects of a person's lifestyle or environment, such as smoking, can increase their chance of developing rheumatoid arthritis. In some people a blood test can show that their immune system has begun to target their own body cells. This increases their chances of going on to develop rheumatoid arthritis. In addition, some people may have symptoms, such as joint pain and stiffness, which increase their chance of developing rheumatoid arthritis.

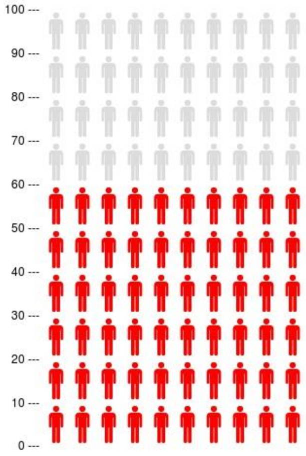

Taken together, these factors can increase a person’s chances of developing rheumatoid arthritis to as much as 60%. In other words, sixty out of every hundred people with these risk factors will develop rheumatoid arthritis, as illustrated by the 60 red figures in this picture. The 40 uncoloured figures represent people with these risk factors who will not develop rheumatoid arthritis.

Treatments for rheumatoid arthritis work best when they are started very soon (within three months) after rheumatoid arthritis has developed. This has led to the idea that **if treatment were started even before someone is diagnosed with rheumatoid arthritis, it may be possible to prevent them from developing the disease altogether.** Researchers are currently trying to develop treatments to reduce someone’s chance of developing this condition. It is important to understand the factors that would influence someone’s decision about whether or not to take such a treatment to reduce their chance of developing rheumatoid arthritis in the future.

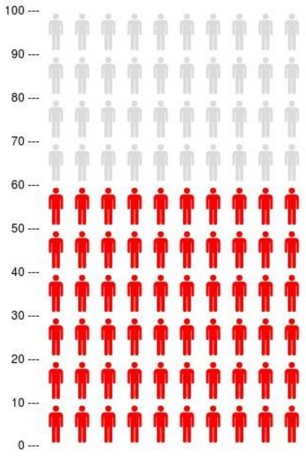

In this survey we will describe some treatments to reduce the chance of developing rheumatoid arthritis. We will ask you to choose the treatment you prefer. Treatment would be taken for one year. When you are making your choice, please imagine that you are experiencing some joint symptoms (joint pain and stiffness) which are starting to impact on your daily activities, and that the results of a blood test show that **you have a 60% chance of developing rheumatoid arthritis in the next 2 years**. Sixty (illustrated by the red figures in the picture) out of 100 people with similar symptoms and blood results will

develop rheumatoid arthritis in the next 2 years, but 40 (illustrated by the uncoloured figures in the picture) out of 100 individuals with similar symptoms and blood results will not.

Survey introduction to treatment attributes and levels

1. Treatment effectiveness

The effectiveness of the treatments varies. The most effective treatment reduces the chance of developing rheumatoid arthritis from 60% (60 in 100), to 10% (10 in 100). This means that only 10 persons in every 100 treated would go on to develop rheumatoid arthritis.
Less effective preventive treatments reduce the chance of developing rheumatoid arthritis from 60% to 40%. This means that 40% (40 persons in 100) would go on to develop rheumatoid arthritis.

2. How the treatment is taken

The available treatments vary in the way they are taken. They include

- An injection: this is a shallow injection under your skin, similar to the way insulin is administered by someone with diabetes. This will be either administered at home or, for some treatments, in hospital.
- A drip: this is an infusion through a hollow needle into the vein at the hospital.
- Tablets: you can take these tablets by swallowing them with some water.

3. How often the medication has to be taken

The available treatments vary in how frequently they are taken. Later in the survey we will ask you to think about preventive treatment that you will have to take from twice a year through to a dose every day.

4. Chance of a mild side effect

The treatments may have some mild side effects which are reversible, meaning they will stop once the treatment stops. These differ between people and could include a low level of nausea, skin rashes or muscle pain.

Later in the survey we will ask you to think about treatments that have a chance of mild side-effects affecting from 2% (2 in 100 people) up to 10% (10 in 100 people). Two percent or 2 in 100 people means that of 100 people who take the treatment, 2 people get these mild side effects while the other 98 people do not.

5. Chance of a serious infection due to treatment

Sometimes the treatment being used to prevent rheumatoid arthritis, can increase your chance of a serious infection, such as pneumonia. Pneumonia is an infection of the lungs. The symptoms include a cough, difficulty breathing, a high temperature, chest pain, and generally feeling unwell. In some cases pneumonia can lead to hospitalisation. The chance of a serious infection varies per treatment. Later in the survey we will ask you to think about treatments that have a chance of a serious infection ranging from no chance of such infection to 5% (5 in 100 people). Five percent or 5 in 100 people means that of 100 people who take the treatment, 5 people get a serious infection while the other 95 people do not.

6. Chance of a serious side effect that is potentially irreversible

In very rare cases, the treatments can lead to serious side effects that potentially might not go away after treatment stops. These include brain inflammation; lymphoma (a type of blood cancer); and retinopathy (potentially irreversible damage to the eye resulting in visual impairment). Later in the survey we will ask you to think about treatments that have a chance of a serious and potentially irreversible side effect ranging from 0.001% (1 in 100,000 people) to 0.1% (100 in 100,000 people). A chance of 1 in 100,000 is similar to one person in a full Wembley stadium getting one of these side effects, if everybody in the stadium was taking the treatment.

Sample PTT choice task:

Given your chance of developing RA in the next 2 years, your doctor suggests that you consider taking a treatment for 1 year. Based on the information presented in the table below, please tell us whether you would choose to take a preventive treatment or if you prefer not to take the preventive treatment by checking the box below your choice.

We will refer to this as your baseline.

	Treatment	No Treatment
**Chance of developing RA [?]**	20%<br>(20 in 100 people)<br>[?]	60%<br>(60 in 100 people)<br>[?]
**Chance of mild side effects [?]**	5%<br>(5 in 100 people)<br>[?]	None<br>(0 in 100 people)
**Chance of a serious infection due to treatment [?]**	2%<br>(2 in 100 people)<br>[?]	None<br>(0 in 100 people)
**Chance of a serious side effect [?]**	0.02%<br>(20 in 100,000 people)<br>[?]	None<br>(0 in 100,000 people)
**I would prefer**	○	○

next

Distribution of maximum acceptable risk intervals for mild side effects

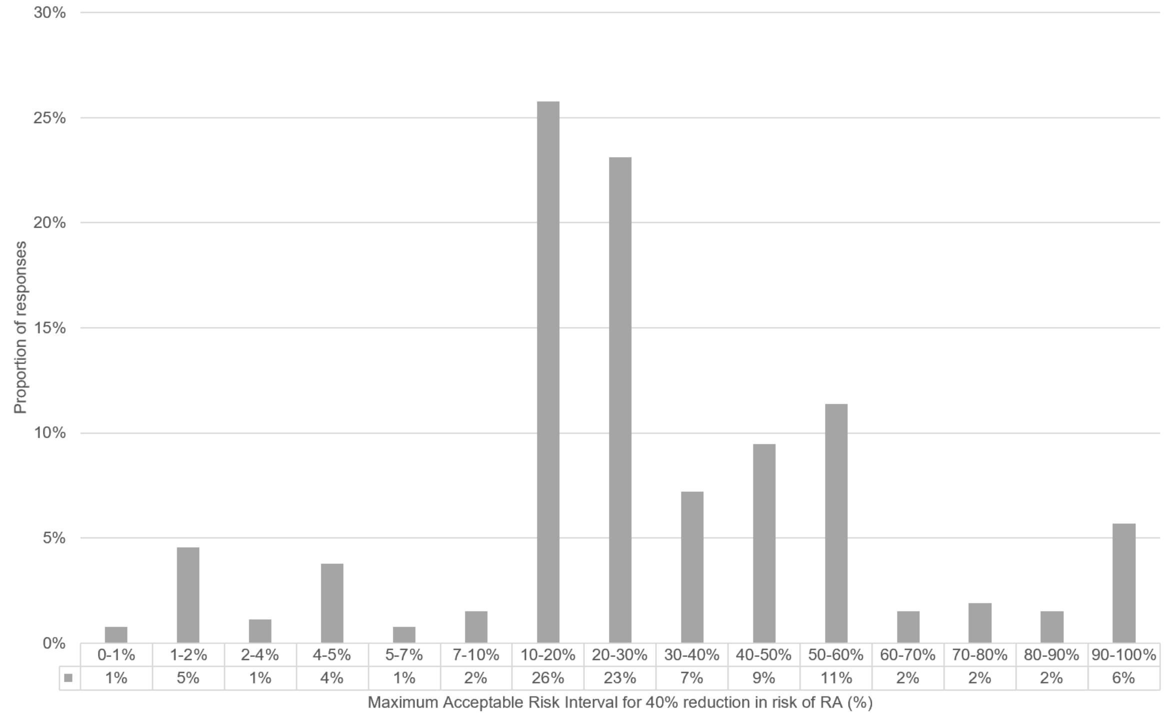

Distribution of maximum acceptable risk intervals for serious infection

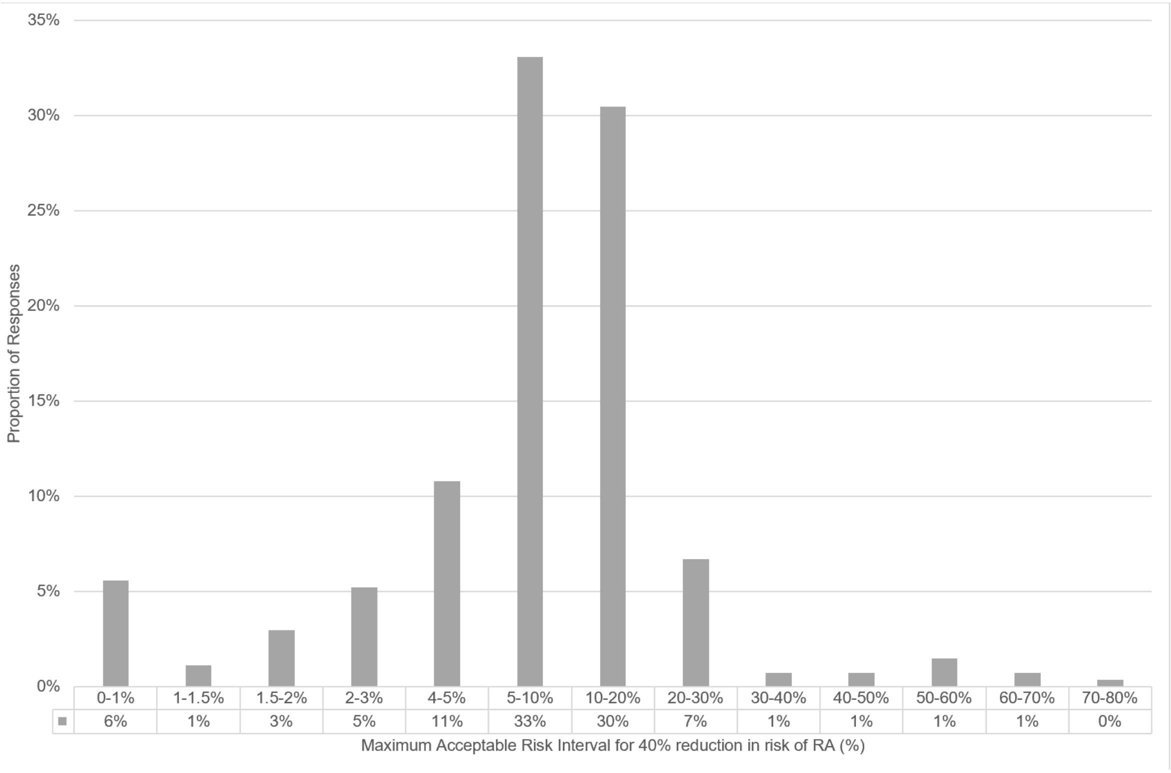

Distribution of maximum acceptable risk intervals for serious side effects

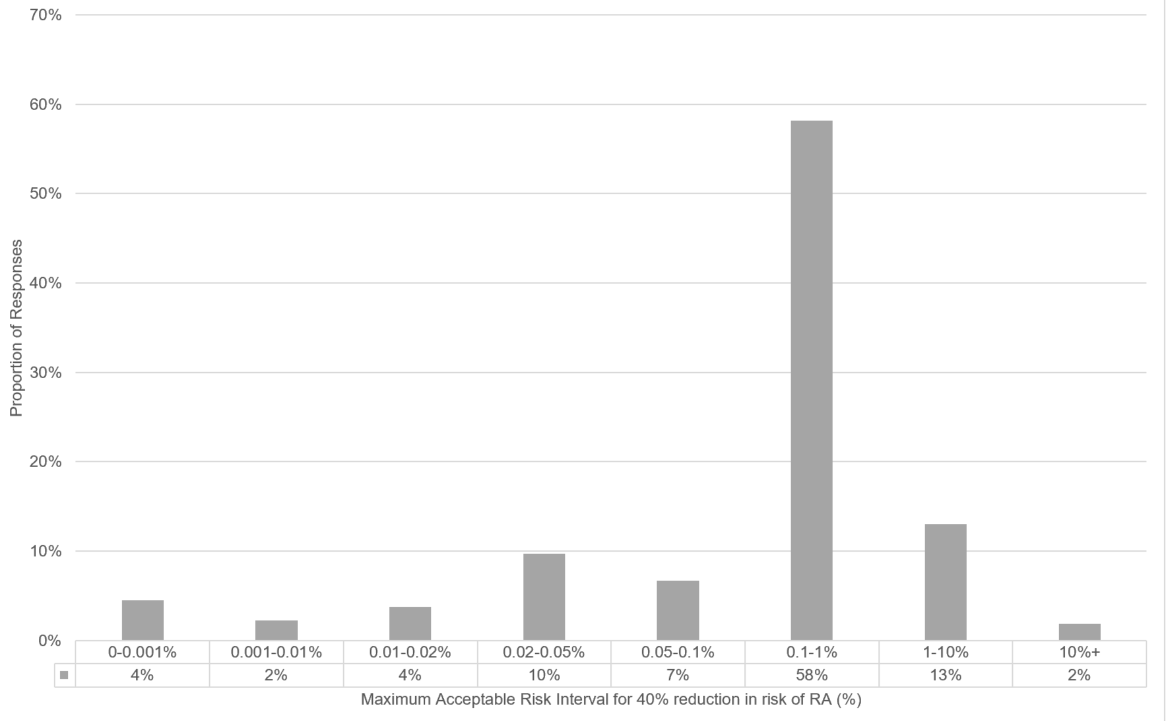

1. Morris NS, MacLean CD, Chew LD, Littenberg B. The Single Item Literacy Screener: evaluation of a brief instrument to identify limited reading ability. BMC Fam Pract. 2006;7:21.
2. McNaughton CD, Cavanaugh KL, Kripalani S, Rothman RL, Wallston KA. Validation of a Short, 3-Item Version of the Subjective Numeracy Scale. Medical Decision Making. 2015;35(8):932-6.
3. Horne R, Weinman J, Hankins M. The beliefs about medicines questionnaire: The development and evaluation of a new method for assessing the cognitive representation of medication. Psychology & Health. 1999;14(1):1-24.
4. Broadbent E, Petrie KJ, Main J, Weinman J. The Brief Illness Perception Questionnaire. Journal of Psychosomatic Research. 2006;60(6):631-7.
5. Broadbent E, Wilkes C, Koschwanez H, Weinman J, Norton S, Petrie KJ. A systematic review and meta-analysis of the Brief Illness Perception Questionnaire. Psychol Health. 2015;30(11):1361-85.
6. Figueiras MJ, Alves NC. Lay perceptions of serious illnesses: An adapted version of the Revised Illness Perception Questionnaire (IPQ-R) for healthy people. Psychology & Health. 2007;22(2):143-58.
7. Russo S, Jongerius C, Faccio F, Pizzoli SFM, Pinto CA, Veldwijk J, et al. Understanding Patients' Preferences: A Systematic Review of Psychological Instruments Used in Patients' Preference and Decision Studies. Value in health : the journal of the International Society for Pharmacoeconomics and Outcomes Research. 2019;22(4):491-501.
8. Russo S, Monzani D, Pinto CA, Vergani L, Marton G, Falahee M, et al. Taking into Account Patient Preferences: A Consensus Study on the Assessment of Psychological Dimensions Within Patient Preference Studies. Patient Prefer Adherence. 2021;15:1331-45.
9. Towards Early diagnosis and biomarker validation in Arthritis Management. Final report. FP7-HEALTH project ID 305549. https://cordis.europa.eu/project/id/305549/reporting.
